# Substation Inspection Safety Risk Identification Based on Synthetic Data and Spatiotemporal Action Detection

**DOI:** 10.3390/s25092720

**Published:** 2025-04-25

**Authors:** Chengcheng Liu, Weihua Zhang, Weijin Xu, Bo Lu, Weijie Li, Xuefeng Zhao

**Affiliations:** 1 State Key Laboratory of Coastal and Offshore Engineering, School of Civil Engineering, Dalian University of Technology, Dalian 116024, China; 21706065@mail.dlut.edu.cn (C.L.);; 2 State Grid Changchun Power Supply Company, Changchun 130021, China

**Keywords:** synthetic data, spatiotemporal action detection, safety risk, Vision Transformer, condition monitoring, optical images

## Abstract

During substation inspection, operators are often exposed to hazardous working environments. It is necessary to use visual sensors to determine work status and perform action detection to distinguish between normal and dangerous actions in order to ensure the safety of operators. However, due to information security, privacy protection, and the rarity of dangerous scenarios, there is a scarcity of related visual action datasets. To address this issue, this study first introduces a virtual work platform, which includes a controller for the parameterized control of scenarios and human resources. It can simulate realistic substation inspection operations and generate synthetic action datasets using domain randomization and behavior tree logic. Subsequently, a spatiotemporal action detection algorithm is utilized for action detection, employing YOLOv8 as the human detector, Vision Transformer as the backbone network, and SlowFast as the action detection architecture. Model training is conducted using three datasets: a real dataset, a synthetic dataset generated via a VWP, and a mixed dataset comprising both real and synthetic data. Finally, using the model trained on the real dataset as a baseline, the evaluation results on the test set shows that the use of synthetic datasets in training improves the model’s average precision by up to 10.7%, with a maximum average precision of 73.61%. This demonstrates the feasibility, effectiveness, and robustness of synthetic data.

## 1. Introduction

With the increasing demand for electricity in recent years, the problem of ensuring high-quality and reliable power has become more urgent. As an important part of power infrastructure, substations require frequent inspection to ensure reliable power supply to cities. Substation operators are often exposed to hazardous working conditions, and safety violations are common due to the impact of the work environment and personal safety awareness. This leads to frequent power safety accidents, which cause huge economic losses and have a negative social impact [1,2,3,4]. Video surveillance systems composed of various visual sensors have become a core technological means of detecting dangerous actions and ensuring public safety in modern society, leveraging their capabilities in multimodal perception, intelligent analysis, and all-weather responsiveness.

In the field of action recognition for substation inspection operations, T. Wang et al. [5] proposed a human climbing fence detection method based on image processing. W. Bo et al. [6] proposed a skeleton-based behavior recognition method to monitor operator violations in distribution network operation. J. Wang et al. [7] combined the YOLOv5-based object detection model, HRNet-based pose estimation model, and ST-GCN-based skeleton-based action recognition model to develop a violation detection strategy for identifying violation behaviors.

In the realm of action detection in machine vision, these studies utilize diverse algorithms for image and skeletal recognition, yet they are often constrained by the proprietary datasets collected by the researchers themselves, which gives rise to several issues. Firstly, there is the issue of dataset imbalance. While it is relatively straightforward to collect data on safe actions, as everyday surveillance can provide ample examples, the collection of hazardous actions poses a challenge. Hazardous actions are rarely observed in daily scenarios, and there are inherent safety risks associated with their collection, leading to a scarcity of such data and an imbalanced dataset [8]. Secondly, the highly specialized field of action detection lacks unified standards for cross-comparison [9]. On the one hand, due to privacy protection and data security limitations, researchers generally use internal video datasets to study highly specialized unconventional activities such as substation inspections. On the other hand, data annotation relies heavily on manual labor, resulting in inconsistent annotation standards and hindering data comparability across studies. The third issue pertains to the fragility of single datasets. Research on the detection and analysis of operator actions during substation inspections under various conditions is relatively limited. Access to video data of operational actions in specialized fields is challenging, and individual videos lack a diversity of different environments, lighting conditions, and multi-angle perspectives of actions in various states. This limitation hinders the study of algorithmic generalizability and robustness.

To solve the problem of insufficient data acquisition by visual sensors in real scenarios, we proposed a method of using virtual technology to simulate engineering operation scenarios and create synthetic datasets for action detection. We introduced a virtual work platform (VWP) to control the generation of synthetic action datasets. We conducted experiments using the VWP in the context of identifying actions during power substation inspection operations. The primary tasks completed are as follows:

(1) We established a VWP. To simulate real-world work scenarios, the VWP supports the import of environmental models and virtual characters and enables the creation of domain randomization controllers, behavior trees, and data export pipelines. By adjusting various parameters and action logic and employing a unified annotation standard, the VWP can generate rich and detailed action datasets with domain randomization, extending to engineering action recognition studies across different domains.
(2) We collected and produced real datasets, synthetic datasets, and mixed datasets for the recognition of safety-risk actions in substations. Upon acquiring these datasets, a thorough comparison of various classical object detection algorithms was conducted. YOLOv8 was ultimately selected as the human detector due to its superior accuracy, rapid detection speed, and lightweight architecture. Furthermore, Vision Transformer (ViT) was integrated as the backbone of the model to effectively extract spatiotemporal contextual information from video sequences, enabling precise action detection by accurately locating the spatial and temporal states of operators. To ensure optimal performance, the model was fine-tuned, optimizing its parameters and achieving the best possible performance metrics.
(3) We used models trained on real datasets as the baseline for comparative experiments, assessing the role and impact of synthetic data through test sets combining real data and AVA data. The results confirmed the feasibility, effectiveness, and robustness of synthetic datasets. Synthetic datasets were found to effectively alleviate the issues of small and insufficiently diverse real datasets, enhancing the accuracy and robustness of the models.

The rest of the paper is organized as follows: Section 2 reviews related work on synthetic data and action recognition. Section 3 describes the composition and architecture of the VWP. Section 4 presents the experimental methodology and results. Section 5 concludes the paper.

## 2. Related Work

### 2.1. Synthetic Data

In traditional machine vision, human action recognition models are trained using large sets of human-labeled data, for example, UCF101 [10], Kinetics 400 [11], HMDB51 [12], AVA [13], Something-Something [14], and other datasets. These datasets, which come from sources such as YouTube, television channels, TV series, and movies, are powerful resources that contain various categories of actions. However, there are the following shortcomings: First, personnel with relevant knowledge are required to manually annotate videos, which is costly, and the annotation will be affected by the subjective judgment of the video annotator. If there are more annotators, the labeling or time judgment of action annotation may be different. Second, the viewing angle of the video in the dataset is limited by the shooting angle, and it cannot be converted, nor can another viewing angle be added according to the requirements of the researchers. Thirdly, in certain professional domains where there is a lack of extensive video data or limited publicly available video data, such as power maintenance, construction, and factory operations, there is a shortage of comprehensive datasets with a wide range of content compared to those available for other types of actions, such as daily activities, dance, or sports movements. Due to concerns regarding information security and privacy, researchers within certain professional domains typically annotate internal video materials for their studies, resulting in fewer publicly released self-created datasets. This situation hampers the ability of researchers in the same field to conduct cross-comparative studies on data of interest.

To address challenges such as limited availability and difficulty in acquiring real-world data, many domains have embarked on a series of research efforts to obtain a larger pool of relevant data and establish more targeted datasets. These endeavors have yielded significant progress.

Among them, the development of simulation experiments in the field of autonomous driving stands out as the most advanced. Several simulation platforms have been developed based on the Unreal Engine game engine, such as Carla [15], developed by Intel Labs, and AirSim [16], developed by Microsoft Research. Additionally, there is a synthetic video dataset called virtual KITTI [17], developed using the Unity game engine. Additionally, there is VIPER [18], developed based on the game Grand Theft Auto V (GTA 5), Synscapes street scene dataset [19], developed based on computer graphics, and a pedestrian trajectory dataset on road network structures, based on digital twins [20]. These virtual simulation open-source platforms and synthetic datasets are designed to support the development of urban autonomous driving systems. They provide accessible digital assets (city layouts, buildings, and vehicles) for 2D and 3D multi-object tracking, with pixel-level labels for categories, instances, flow, and depth. These resources serve as valuable tools for computer vision tasks related to urban autonomous vehicle navigation and contribute to the advancement of research in autonomous driving.

In the field of object pose detection, research into synthetic datasets for the purpose of expanding data collections and enhancing model generalization capabilities has been ongoing. Tremblay et al. introduced a synthetic dataset called Falling Things (FAT) [21]. This dataset provides 3D poses for all objects, pixel-wise class segmentation, and 2D/3D bounding box coordinates. NVIDIA’s Deep Learning Dataset Synthesizer (NDDS) plugin supports image, segmentation, depth, object pose, bounding box, keypoints, and custom template synthesis. It includes the randomization of lighting, objects, camera positions, poses, textures, and occlusions, as well as camera path tracking. Unity’s Perception package [22] provides a tool for capturing datasets, allowing the merging and generation of synthetic datasets within Unity. It encompasses four fundamental functionalities: object labeling, annotation, image capture, and customizable metrics.

In the field of pedestrian detection and tracking, Fabbri M [23] collected the JTA (Joint Track Auto) dataset and the MOTSynth [24] dataset for pedestrian pose estimation and tracking in urban scenarios by exploiting the highly photorealistic video game GTA 5. Hu Y T et al. also utilized the game GTA 5 to generate the SAIL-VOS [25] dataset, which consists of instance-level video object segmentation with occlusions. Additionally, they introduced SAIL-VOS 3D [26], an extended synthetic video dataset with per-frame grid annotations. Unity introduced PeopleSansPeople [27], a parameterized synthetic data generator for human recognition. It allows the generation of synthetic data with varying poses, lighting conditions, positions, and occlusions to enhance human recognition tasks.

Most of the research methods described above use game engines or build data simulators based on commercial games with high completion rates. The simulators provide control over the data generation itself and facilitate the adjustment of datasets for simulation-to-reality transfer. The generated data types typically include images, videos, and radar data. This proves the value of using the simulator to synthesize datasets in the research fields of image processing [28], object pose detection [29], person detection [30], trajectory tracking [31], automatic driving [32,33], dangerous scene detection, and so on. However, these studies do not allow researchers to import, control, or set the behavior logic of the characters. Inspired by the above research, we built a virtual work platform (VWP) based on Unreal Engine. The VWP can control the parameter distribution of light, people, action, camera, etc., and the action logic simulates the operation of real substation inspection personnel, providing a solution for researchers to address the lack of relevant action datasets when studying highly professional action detection such as substation inspection.

### 2.2. Action Detection

Research on action recognition can be roughly divided into three directions. The first direction is action recognition, which can only be applied to cropped videos to judge the action category. The video containing the recognized action needs to be classified and labeled with action labels after being cropped, and each video in this category contains only one type of action. The second direction is temporal action detection, which targets the original video that has not been cropped, and can locate the time of the action, giving the start and end time points of the action and the action category information in the video. However, temporal action detection can only detect and locate actions in the temporal domain. In reality, the situation is usually more complex, as practical scenarios may involve multiple people and multiple types of action categories at the same time. To address the complexity of real-world scenarios, the third direction, spatiotemporal action detection [34], further locates the action in both the temporal and spatial domains to solve the problem of multiple people and multiple actions in complex scenes. Specifically, it can not only provide the action category and start/end time points but also locate the spatial region where the action occurs, as well as tracking personnel between different frames in the time stream.

To analyze the operation processes of inspection operators in substations, it is necessary to detect the location of the operators in both the spatial and temporal domains. Therefore, we use spatiotemporal action detection to obtain the occurrence time, category, and spatial position of the operators during their operations.

Frame-Level Action Detection. The advancement of image object detection technology has inspired frame-level action detection methods. Frame-level action detection is an action detection method based on frame images, which can be mainly divided into two stages. First, frame-level action proposal detection is generated through region proposal algorithms. Then, proposals are associated across frames to refine action classification and localization, enabling the localization and detection of actions over time [35,36]. Methods for action detection include traditional feature extraction methods, such as generating spatiotemporal shapes using super-voxels to provide 2D + t sequences, or obtaining feature points through dense sampling [37,38,39], whereas dense trajectory methods [40,41,42] extract features based on trajectory tracking of feature points and then classify proposals to locate actions. There are also feature extraction methods based on deep learning [43,44,45,46], which capture action features using optical flow and predict action categories using convolutional neural networks (CNNs), region proposal networks (RPNs), Fast R-CNNs, and 3D convolutional neural networks and connect frame-level bounding boxes into the spatiotemporal action tubes. They also capture spatial and static visual cues using RGB images and extract time and action information from optical flow fields for action estimation [47]. The main drawback of frame-level action detection is that because detection is performed independently in each frame, the video’s temporal information is not fully utilized [48].

Clip-Level Action Detection. Clip-level action detection is crucial for accurately recognizing many types of actions, such as sitting down, standing up, picking something up, and putting something down, which cannot be identified based on individual frames alone. This is because effective temporal modeling is required, as the detection of such actions relies on the availability of temporal context. Kalogeiton et al. [49] proposed a tubelet detector that leverages the temporal continuity of video frames to detect objects in sequences. While this approach effectively utilizes temporal information, it faces several challenges, including computational complexity, difficulties in handling complex scenes, demanding training requirements, and limited contextual understanding. Gu et al. [13] proposed an atomic visual action (AVA) dataset that has precise spatiotemporal annotations and dense annotations for atomic visual actions. They proposed an action detection model based on I3D convolution and Faster R-CNN region proposals and demonstrated the importance of time information by comparing the performance of edited videos of different time lengths on the model. However, the employed Faster R-CNN model is highly complex, resulting in relatively slow detection speeds. Additionally, I3D is limited by its fixed temporal window and local convolution operations, which restrict its ability to capture long-range contextual information. Feichtenhofer et al. [50] established a SlowFast network model for video action detection, which employs ResNet as the backbone and captures spatial semantics at low frame rates and action information at high frame rates. Nevertheless, the utilized ResNet backbone network has difficulty capturing global dependencies and shows limited performance gains on large-scale datasets. Recently, Transformer [51] has made significant advances in the field of natural language processing [52]. Tasks such as image classification, object detection, semantic segmentation, object tracking, and video recognition in the field of computer vision have also been improved through the use of Vision Transformer (ViT) [53]. Based on the development of ViT, Tong Z et al. [54] proposed a self-supervised video pre-training method, called video masked autoencoders (VideoMAE).

Based on the VideoMAE algorithm, which utilizes SlowFast as the framework and ViT as the backbone network to effectively capture long-range dependencies and compute global dependencies, we selected YOLOv8 as the human detector. We employed the flexible and scalable architecture of ViT, along with its strong pre-trained performance, to conduct transfer learning for action detection. To better leverage long-range information between frames, we adopted the clip-level action detection method and generated synthetic data in the AVA format via VWP for spatiotemporal action detection.

## 3. VWP

In Section 2, we discussed the landscape of synthetic data in the context of action recognition research and introduced our virtual work platform (VWP) for synthetic data generation. In this section, we detail the components of our synthetic data generator, the VWP.

**Figure 1:**
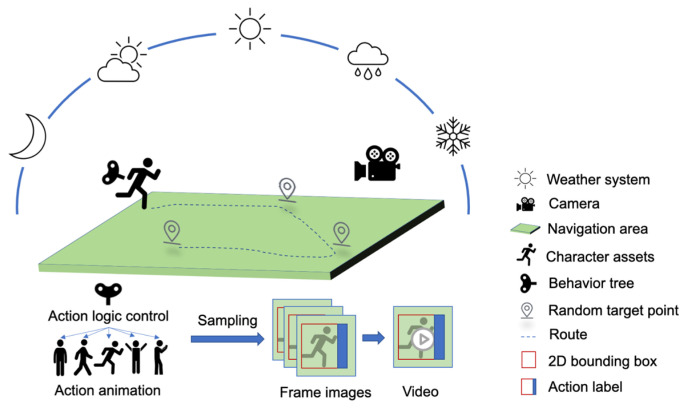
Composition framework of VWP.

We utilized the powerful editing capabilities of the Unreal Engine game engine to create the virtual work platform (VWP), which is a parameterized data generator with a 3D scene of a substation, 3D models of operators with clothing, skeletons, and action libraries, and support for importing new character models and actions. The VWP supports the generation of data with 2D bounding box positions of operators and corresponding action labels, and outputs data in the format of the AVA dataset, with the duration and frame rate of the output video being adjustable through parameters. The composition framework of the VWP is shown in Figure 1.

### 3.1. Character Assets

**Figure 2:**
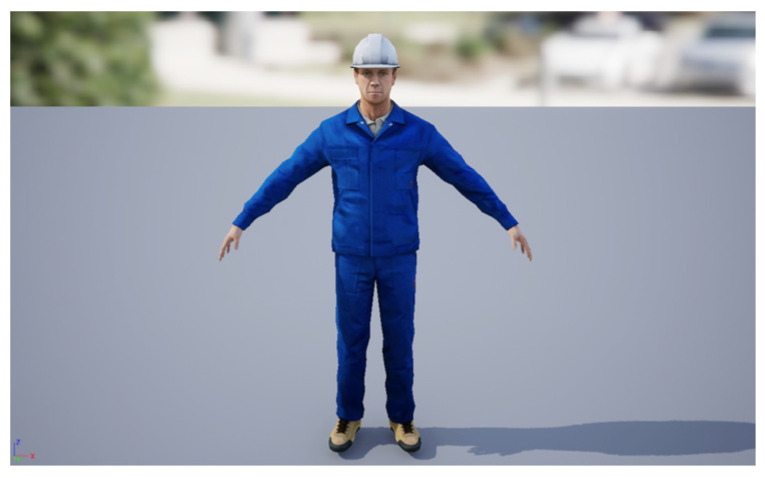
Virtual avatar of operator.

The VWP imported Microsoft-Rocketbox [55], an open-source character library from Microsoft, which provides virtual avatars with built-in skeletons and covers different ages, genders, skin tones, clothing, and professions. For our experiment, we selected the model in Microsoft-Rocketbox wearing an operator uniform to simulate substation inspection operators, as shown in Figure 2.

### 3.2. Action Animation

In our experiments, the actions selected for power substation inspection operations include standing, walking, running, waving, and making phone calls. Among these, standing and walking are considered normal behaviors, while running, waving, and making phone calls are regarded as violations. In the field of action recognition, the quality of the dataset plays a crucial role in the performance and accuracy of the model. We achieve the playback of action animations and the acquisition of action data in the VWP through the following three steps:

Step one involves the selection of action data. To ensure that the model possesses a certain degree of generalization capability, the dataset should encompass a diverse range of samples. The actions utilized in this study were sourced from multiple action libraries: the CMU Graphics Lab’s CMU Motion Capture Database [56], the action library from Microsoft’s Rocketbox, and real human actions captured on video using ROMP pose recognition technology. Each action label comprised three distinct actions.

**Figure 3:**
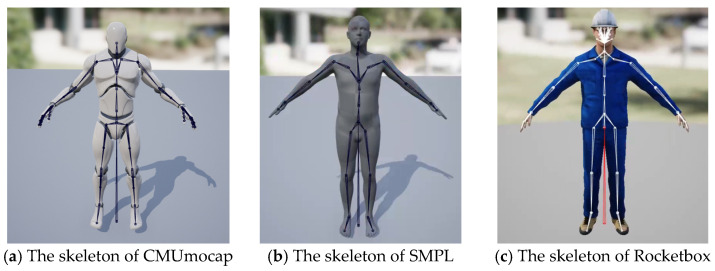
The skeleton of different models.

Step two pertains to the representation of action data. The skeleton of the CMUmocap dataset, the SMPL model used in the ROMP algorithm, and the Microsoft-Rocketbox action library are shown in Figure 3a–c, respectively. This prevents data from various motion libraries from being uniformly applied to the UE character. In order to tackle this problem, we employed inverse kinematics to retarget the actions, aligning them to various skeletons and facilitating the transformation of action animations from different action library models into a unified skeleton model. Once the action was unified across the skeleton models, within the Unreal Engine, we controlled the character model’s movements by playing back animations and enriched data acquisition by setting various playback speeds, yielding action data at different velocities.

Step three is the annotation of action data, where accurate labeling is a prerequisite for training effective models. For each action label, we first selected multiple corresponding distinct action animations and established a mapping relationship between the action animations and the action labels. Subsequently, we sampled the state of the played-back action animations to obtain action label data. Finally, the VWP could automatically generate labels by setting the sampling frequency of the action labels according to custom parameters. Here, we adopted the AVA data format to generate datasets, producing one action label per second.

### 3.3. Action Logic Control

**Figure 4:**
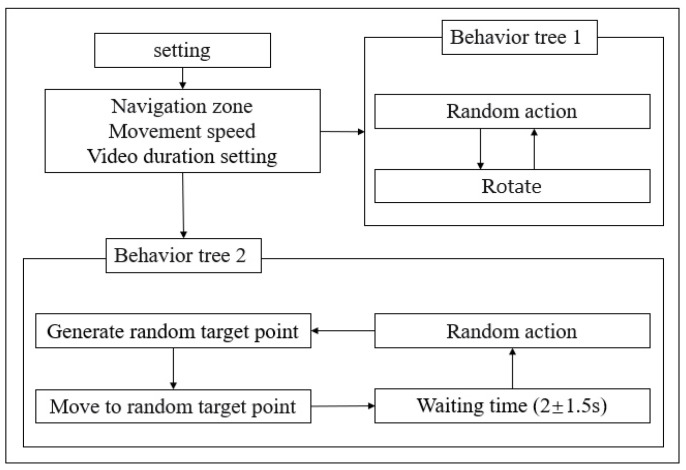
Behavior tree logic architecture.

With the basis of the action animation, we also aimed to control the playback of these animations according to a certain logic to better conform to the movement logic of real people. So, when creating the experimental dataset, we established operator behavior trees to control the conversion and connection of various actions. There were mainly two different behavior trees, which are shown in Figure 4. The first behavior tree was designed to have characters perform random actions and collect action data from multiple angles. After the termination of one action, the character rotated by an arbitrary angle (here, we set it to 15°) before proceeding to the next action. The second type was based on the action behavior logic of power substation inspection operations. Firstly, a navigation area of 4 m × 10 m was set as the inspection operator’s movement area, and a random target point was set as the inspection checkpoint. The operator reached the target point by walking or running and then took a 2.0 s (±1.5 s; randomly selected between 0.5 and 3.5 s) standing rest, before performing random actions from the action library such as standing, waving, or talking on the phone. The operator then moved on to the next target point and repeated the above actions. Then, we continued to the next target point and repeated the above actions.

### 3.4. Background Model

**Figure 5:**
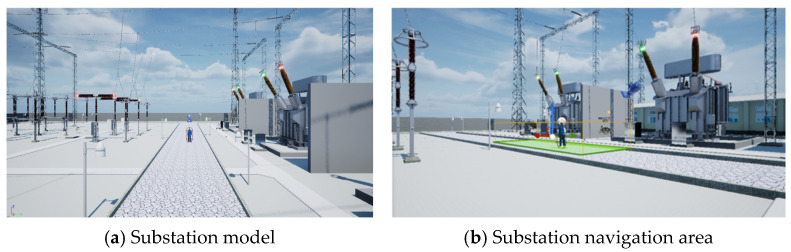
Background model.

We studied action detection for substation inspection operations, so the substation model was chosen as the background model. The scene was set to an outdoor environment, with the navigation area located on the sidewalk of the substation. The experimental area is shown in Figure 5, where (a) displays the substation model and (b) shows the navigation area highlighted in green, indicating the area where operators can move during the experiment.

### 3.5. Weather System

The VWP uses the weather plugin of Unreal Engine to control weather and lighting. The weather system can modulate lighting to simulate the variations in light throughout different times of the day, and can adjust wind, clouds, rain, and snow to simulate various weather conditions.

### 3.6. Data Acquisition

In the VWP, the acquisition interval for 2D bounding box data of the human body can be set to achieve frame-by-frame acquisition. In our experiment, we followed the format of the AVA dataset and obtained 2D bounding box data of the human body every second. We detected the played animation every second, mapped the animation to the corresponding action label, obtained the action label data, and saved each frame of the image as video data.

### 3.7. Domain Randomization

Domain randomization [57] is a technique that introduces diversity into generating data by randomizing the parameters of a simulator. Domain randomization has been applied to tasks including object detection, robot manipulation, autonomous vehicle navigation, and other types [27]. In order to extend our synthesized dataset to simulate the real world, we randomized multiple parameters in the environment.

In the experiment, we controlled the climate environment, shadows, action animation playback speed, character position, and camera deployment parameters in the VWP and used uniform distribution to sample the parameters to generate a dataset. The distribution of parameters in the VWP-generated dataset is shown in Table 1.

In this section, we introduce the various components of the VWP framework and the method for generating synthetic data. Through the VWP, we have obtained a rich collection of synthetic data. In Section 4, we will utilize this synthetic data to train action detection models and validate the effectiveness of the synthetic data by comparing the performance of models trained on different datasets.

## 4. Experiments

**Figure 6:**
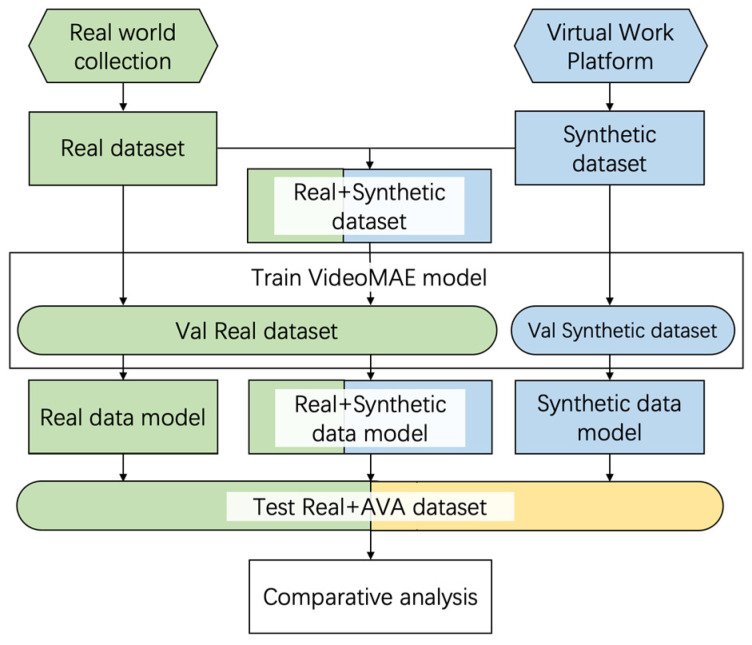
The method of the experiment.

In this section, we conduct experiments using models trained on various datasets with a spatiotemporal action detection algorithm, employing the model trained on the real dataset as a baseline to assess the importance of synthetic datasets in action detection. We used the spatiotemporal action detection method of VideoMAE, which utilizes YOLOv8 as the human detector, ViT as the backbone, and SlowFast as the action detection framework. To facilitate data collection for comparison, we selected five basic and simple actions in substation inspection operations, namely, the normal actions of standing and walking and the violation actions of running, waving, and talking on the phone as categories for spatiotemporal action detection. We collected both real and synthetic datasets containing these actions. Then, we trained models using different datasets and finally tested the models on a real data test set, evaluating five action categories using mean average precision (*mAP*) as the metric. The method of the experiment is depicted in Figure 6. A detailed explanation of the dataset classification and model training is provided in Section 4.2 and Section 4.4, respectively.

### 4.1. Experimental Configuration

The experimental configuration is shown in Table 2.

### 4.2. Data Preparation

**Figure 7:**
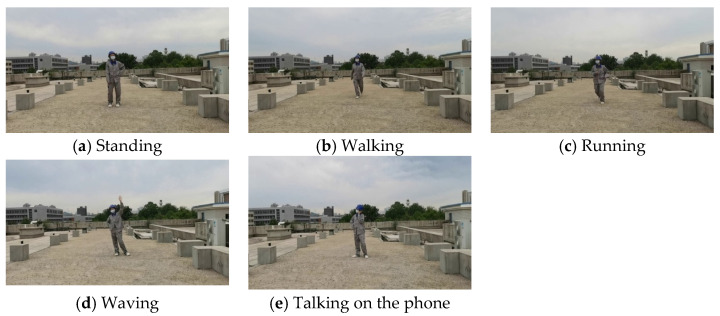
Examples of the real dataset.

Real dataset: We shot videos in a real environment to create a real action dataset. The action categories included five categories: standing, walking, running, waving, and talking on the phone. The training and validation sets of the real dataset only used positive action videos with fixed personnel positions (walking and running positions were not fixed), while the testing set used real video data with more random personnel positions and angles to test the effectiveness of the model. The real dataset consisted of 25 videos captured in real scenes, each with a duration of 1 min. Among them, there were 20 videos in the training set and 5 videos in the validation set. The resolution was 1080 × 720, at 30 frames per second. The real dataset is shown in Figure 7.

**Figure 8:**
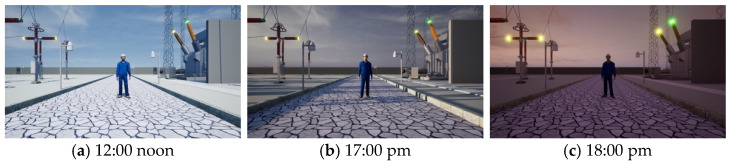
Scenes at different times. Several time periods with significant changes in lighting were selected to obtain data.

**Figure 9:**
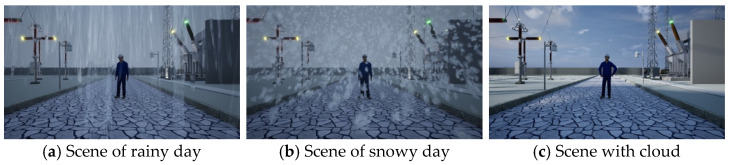
Scenes with different weather conditions.

**Figure 10:**
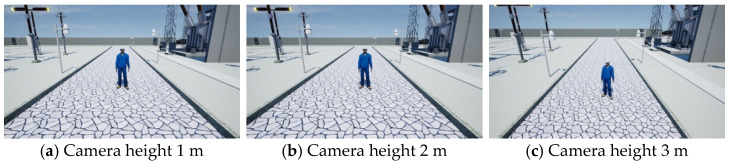
Scenes at different camera heights.

Synthetic dataset: Based on the VWP, we obtained a synthetic video dataset called the v dataset through parameter randomization. The specific parameter adjustments are detailed in Section 3, VWP. We obtained a total of 50 videos through the VWP, each with a duration of 5 min. Among them, there were 40 training videos and 10 validation videos. The resolution was 1080 × 720, at 30 frames per second. To demonstrate the specific performance of the synthetic dataset, we selected keyframe images for display, as shown in Figure 8, Figure 9 and Figure 10.

Test dataset: To assess the accuracy and generalizability of action category recognition, we compiled a test set by combining our collected real-world data with the more diverse AVA dataset.

The collected real-world data consisted of 10 videos captured in real scenes. Each video had a duration of 1 min. The resolution was 1080 × 720, at 30 frames per second. Given that our dataset was limited to 5 action categories, which were fewer than the 80 action categories present in the AVA dataset, and the action categories were not in a one-to-one correspondence, we processed the AVA dataset as follows: 1. Data with inconsistent action types were excluded from testing. 2. Since a single video may contain both action types consistent with our dataset and inconsistent action types, we selected only the bounding boxes of individuals performing consistent actions for testing. 3. As our focus was on action recognition, we modified the human bounding box detection score threshold to 0.9 for the action recognition task.

To further investigate the impact of synthetic data compared to different sizes of real datasets and mixed datasets, we split the training set of the real dataset into subsets and recombined them into four different-sized datasets, namely, real-1, real-2, real-3, and real-4, which were then used for model training. Similarly, we combined the real dataset subsets with the synthetic dataset to form mixed datasets, namely, real-1 + v, real-2 + v, real-3 + v, and real-4 + v, which were also used for model training. In total, there were nine datasets, comprising synthetic, real, and mixed datasets. The specific composition of these nine datasets is shown in Table 3.

Evaluation metrics: In classification problems, the model can produce True Positive (*TP*), False Negative (*FN*), False Positive (*FP*), and True Negative (*TN*) cases during testing. When the confidence score is higher than the threshold, the object is identified as *TP*; otherwise, it is identified as *FP*. *FN* represents the number of undetected objects. *TN* is not considered in classification. Precision (*P*) and recall (*R*) for each class can be calculated using (1) and (2), and the average precision (*AP*) for a single class is evaluated using (3). In this study, as this was a multi-class classification model, mean average precision (*mAP*) was used for evaluation, as shown in (4).

Precision (*P*):

(1) $$P=\frac{TP}{TP+FP}\;$$

Recall (*R*):

(2) $$R=\frac{TP}{TP+FN}\;$$

Average precision (*AP*):

(3) $$AP=\int_{0}^{1}P\left(R\right)d(R)\;$$

Mean average precision (*mAP*):

(4) $$mAP=\frac{1}{n}\sum_{k=1}^{n}{AP}_{k},\;\;\;n=5$$

### 4.3. Experimental Method

In this experiment, we utilize the VideoMAE [54] algorithm to extract spatiotemporal contextual information from videos for action detection. VideoMAE inherits the simple pipeline of masking random cubes and reconstructing the missing ones. This method can successfully train an ordinary ViT backbone on relatively small video datasets and has achieved excellent results on AVA datasets for spatiotemporal action detection.

**Figure 11:**
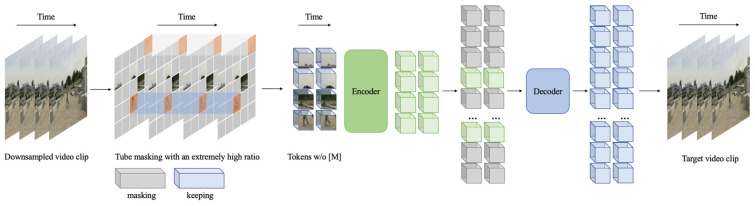
The overall pipeline of VideoMAE.

The advantages of VideoMAE lie in its consideration of the high temporal redundancy and time correlation of videos, which can result in low learning efficiency and information leakage risks compared to images. To address these issues, VideoMAE adopts a stride time sampling strategy and joint space–time cube embedding and uses a very high masking rate to remove masked cubes from downsampled segments, as shown in Figure 11. This not only effectively improves pre-training performance but also significantly reduces computational costs through the use of an asymmetric encoder–decoder structure. VideoMAE is a highly data-efficient learning solution that is suitable for video datasets of different sizes. Additionally, ViT models pre-trained through self-supervised learning on massive datasets exhibit better generalization capabilities on different datasets. ViT models pre-trained on real-world datasets learn implicit features of real-world images, which can aid transfer learning between synthetic and real datasets [53]. Considering the aforementioned advantages, we selected the VideoMAE pre-trained ViT as the backbone of our model to investigate the robustness of synthetic datasets on models with different parameters.

**Figure 12:**
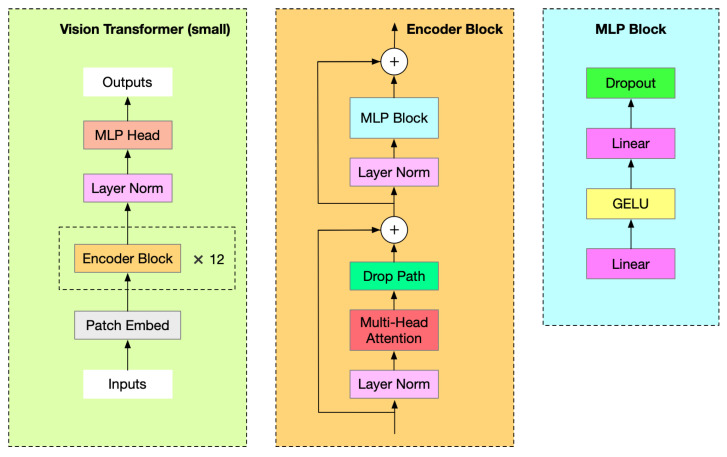
The ViT-small backbone of the spatiotemporal action detection algorithm.

When leveraging the pre-trained ViT model for the specific downstream task of spatiotemporal action detection, there is no need for the decoder block of the ViT model to reconstruct masked video blocks. In this context, an MLP (Multi-Layer Perceptron) head replaces the decoder block, effectively assuming the role of processing the detection task. The specific architecture of the model is illustrated in Figure 8. The process involves four steps: first, the Patch Embed module segments the input video into patches; second, these patches are fed into the Encoder Block for feature extraction; third, Layer Norm normalizes the feature dimensions; and finally, the MLP head fully connects the normalized features to the output features, yielding the final classification outcome. The detailed architectures of the encoder block and MLP block are depicted in Figure 12.

Additionally, in order to rapidly and accurately extract human bounding boxes and enhance the precision of action detection, we conducted experiments on a dataset to compare the performance of various object detection models. We selected classic algorithms in object detection, including the two-stage detection models RCNN and Faster R-CNN [58], and the one-stage detection models RetinaNet [59], YOLOv5 [60], and YOLOv8 [61] for comparison. The experimental dataset was the real-4 dataset, which was collected from real-world scenarios and is used to evaluate the models’ performance in practical settings.

Under the conditions of an IoU threshold of 0.5 and the confidence threshold of 0.8, we recorded the *AP* values and the detection time per frame (milliseconds per frame, ms/f) for each model, with the results presented in Table 4. As shown in Table 4, YOLOv8 achieved an *AP* value of 97.7%, demonstrating the highest detection accuracy among the models tested. Additionally, its average detection time was 18.3 ms/f, which is significantly better than that of the other models. In comparison, YOLOv5 exhibited an *AP* value of 97.2% and a detection time of 19 ms/f, which are close to but slightly inferior to those of YOLOv8. RetinaNet achieved an *AP* value of 91%; however, its detection time of 138.9 ms/f is considerably longer than that of the YOLO algorithms. Both RPN and Faster R-CNN performed poorly in terms of both detection accuracy and detection time.

**Figure 13:**
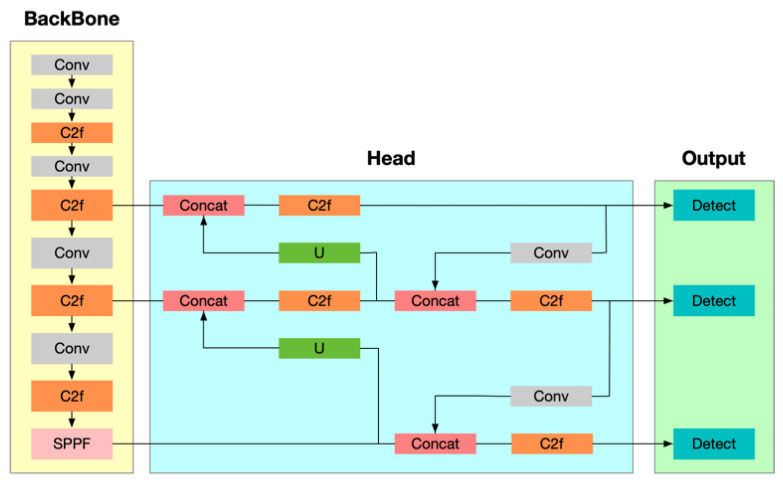
The architecture of YOLOv8.

Considering both the *AP* values and detection times, we selected YOLOv8 as the framework for human bounding box detection. YOLOv8 maintains high detection accuracy while processing images rapidly. Additionally, the framework features a lightweight and scalable design [62,63]. The architecture of YOLOv8 is illustrated in Figure 13. We also followed the classic and effective SlowFast action detection architecture, which captures spatial semantic information at a low frame rate through a slow pathway and action information at a high frame rate through a fast pathway, to achieve spatiotemporal action detection.

### 4.4. Training

To evaluate the performance of synthetic, real, and mixed datasets, we trained the nine datasets on the model. Consistently with our computer configuration, we elected to fine-tune the pre-trained ViT-small model. The training was conducted using the parameters from the VideoMAE [54] paper as a foundation for fine-tuning. We fine-tuned the hyperparameters, including the optimizer, base learning rate, and layer decay, to improve the model’s accuracy. We utilized the real-4 dataset for hyperparameter selection with real data and the real-4 + v dataset for hyperparameter selection involving mixed datasets and virtual datasets. We meticulously tuned these hyperparameters to successfully train the model and to adapt it to the task.

The results of the model fine-tuning are presented in Table 5.

**Figure 14:**
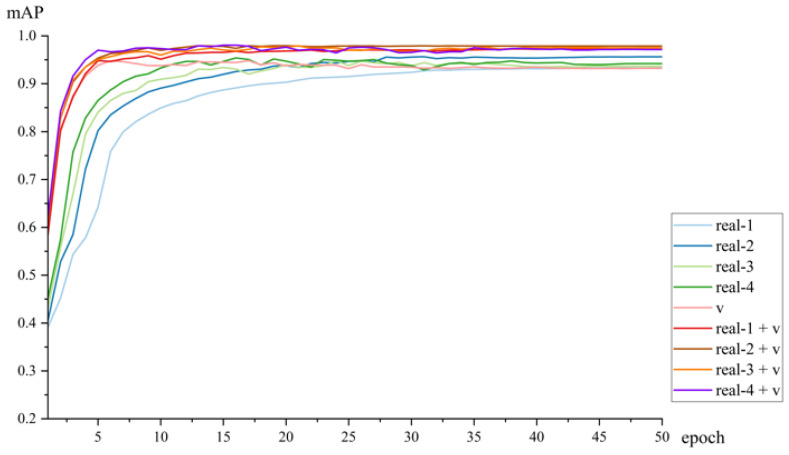
The *mAP* trained on the model for different datasets.

The training process of the model is depicted in Figure 14. Since the validation set for the synthetic dataset consists of synthetic data, we tested the data obtained from training on synthetic data on a real-world validation set. The test results are compared here with those from models trained on real datasets and mixed datasets, where the validation set is uniformly composed of real-world data.

The synthetic dataset v experienced a drop in *mAP* during the middle of training, possibly because the model learned more noise from the synthetic dataset that differs from the real dataset, which weakened the model’s performance on the real val dataset. However, this issue was alleviated when real datasets were added to the synthetic dataset, and the model trained on the mixed dataset showed more stable performance. The model with the highest *mAP* value was trained on the mixed dataset real-4 + v.

The models trained on mixed datasets consistently yielded higher mean *mAP* values than those trained on real datasets, and they demonstrated faster convergence compared to models trained on real datasets alone. Among these, the model trained on the mixed dataset real-4 + v exhibited the highest *mAP* value.

The training results are shown in Table 6, and the highest *mAP* (%) of the model during the training is used as the basis for the model analysis results.

**Figure 15:**
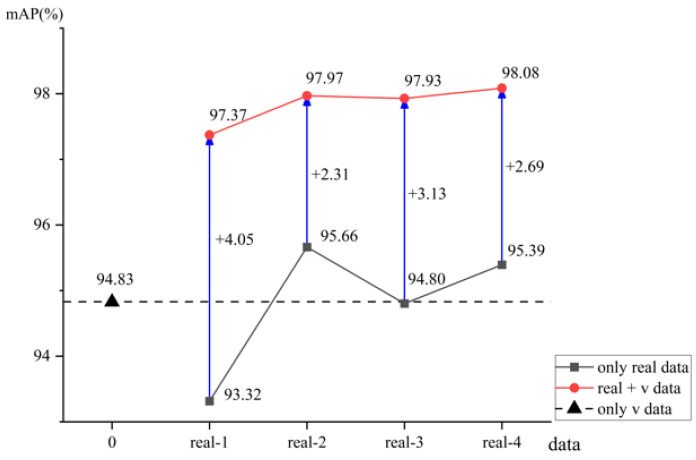
The training performance of different datasets on the model.

In order to better analyze the experimental results, Figure 15 shows a comparison of the model training results.

As detailed in Table 6 and Figure 15, the maximum *mAP* values achieved by models trained on real, synthetic, and mixed datasets were 95.66%, 94.83%, and 98.08%, respectively. The model trained on the synthetic dataset outperformed those trained on the real-1 and real-3 datasets, while it was only 0.83% lower than the highest *mAP* attained by the real-2 dataset. This proves the rationality of using the synthetic dataset to train the model.

The *mAP* values of models trained on real datasets ranging from real-1 to real-4 displayed considerable variation, without a steady upward progression. This fluctuation may stem from the increased sensitivity of models trained on smaller datasets to the randomness inherent in sample selection, resulting in substantial performance discrepancies across different training sets. Notably, while the model trained on the real-2 dataset achieved the highest *mAP* among the real datasets, its performance on the test set, as illustrated in Figure 15, was inferior to that of models trained on the real-3 and real-4 datasets. This suggests potential overfitting on the validation set for the real-2 dataset-trained model.

Models trained on mixed datasets consistently achieved higher *mAP* values than those trained on real datasets, with the highest *mAP* difference reaching 2.42%. Additionally, the maximum and minimum *mAP* values for models trained on real datasets spanned a range of 2.34%, while for mixed datasets, this range was narrower, at 0.71%. The increase in *mAP* values for models trained on mixed datasets was minimal with the addition of more real data, indicating that the volume of real data in the mixed dataset has a limited impact on model performance. Models trained on mixed datasets demonstrated enhanced accuracy and stability.

### 4.5. Results

The test dataset was a composite of real-world data and AVA data. The real-world data were derived from specific scenarios we had collected, whereas the AVA dataset encompassed a broader range of sources, reflecting diverse environments and conditions. This combination test set can better simulate the diverse situations that the model may encounter in practical applications, thereby evaluating the model’s adaptability and generalization performance to different data distributions. By employing this approach, we can ensure that the model not only performs well on specific datasets but also maintains stable performance on a broader spectrum of unseen data, which is crucial for evaluating the model’s generalization ability.

**Figure 16:**
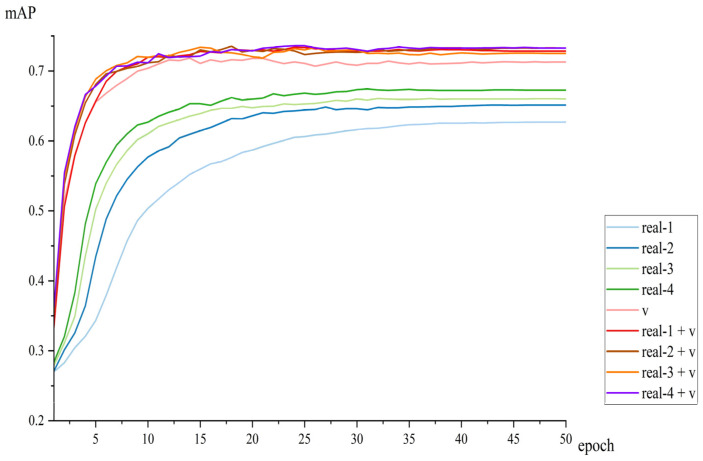
The *mAP* of models trained on different datasets on the test dataset.

The performance of the model across various epochs during the testing phase is depicted in Figure 16. Throughout the testing process, the *mAP* of the model trained on the synthetic dataset v consistently surpassed that of models trained on real datasets, indicating that synthetic data confer superior generalization capabilities compared to models trained exclusively on real datasets. However, a notable decline in *mAP* for the synthetic dataset v was observed in later epochs. This may be because the model learned more noise from the synthetic dataset, thereby weakening its performance on real test datasets. This issue was mitigated by incorporating real datasets into the synthetic dataset; models trained on mixed datasets demonstrated greater stability than those trained on synthetic data alone. Notably, the model trained on the mixed dataset real-4 + v achieved the highest *mAP* value of 73.61. The mixed dataset not only facilitated the fastest convergence but also yielded the highest *mAP*, highlighting its effectiveness in enhancing model performance.

We took the highest *mAP* (%) of the model during the test as the basis for the model analysis results, and the test results are shown in Table 7.

**Figure 17:**
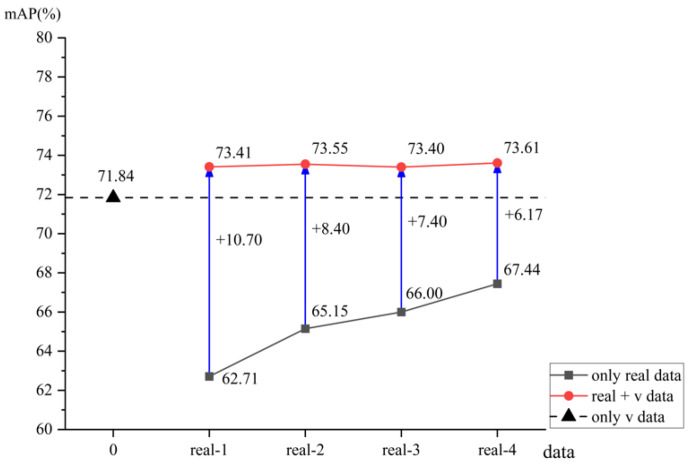
The test performance of different datasets on the model.

In order to better analyze the experimental results, Figure 17 shows a comparison of the model testing results.

From the experimental results of the model in Figure 17, it can be observed that due to the small amount of data, the *mAP* value of the model trained on the real-1 dataset was the lowest. As the data volume increased, the *mAP* values of the model trained on real datasets from real-1 to real-4 gradually increased, with the highest *mAP* value of 67.44 achieved by the model trained on the real-4 dataset. The *mAP* value of the model trained on the synthetic dataset v was 71.84, which was higher than the models trained on all real datasets.

Models trained on mixed datasets ranging from real-1 + v to real-4 + v exhibited *mAP* values that surpassed those of the synthetic dataset v. Notably, the model trained on the real-4 + v dataset achieved the highest *mAP* value of 73.61. The greatest improvement was observed between the model trained on the real-4 + v dataset and that trained on v, with a difference in *mAP* values of 1.77. This indicates that the incorporation of real data into synthetic data significantly enhances the *mAP* performance of mixed datasets during model training.

When comparing models trained on mixed datasets from real-1 + v to real-4 + v, the maximum difference in *mAP* values was observed between real-4 + v and real-3 + v, with a minimal difference of 0.21. In contrast, the models trained on the real datasets from real-1 to real-4 showed a much larger maximum *mAP* difference of 4.73, significantly greater than the 0.21 difference observed in the mixed datasets. Furthermore, as the proportion of real data in the mixed datasets increased, the performance improvement of the models trained on these mixed datasets was marginal, whereas the models trained on the real datasets exhibited substantial improvements. Correspondingly, the *mAP* differences among models trained on real datasets decreased, suggesting that the volume of real data in mixed datasets has a minimal impact on model performance.

The maximum value obtained from models trained on mixed datasets differed by 6.17 from the maximum value achieved by models trained on real datasets, indicating that mixed datasets can enhance the accuracy and generalization capabilities of the models.

### 4.6. Discussion

Based on the comparison of training and testing results of the ViT model on nine datasets, including real, synthetic, and mixed datasets, we discuss and summarize the findings as follows:

(1) Adverse Effects of Small Real Datasets

On one hand, when a real dataset has a limited amount of data, models trained on this dataset are significantly influenced by the randomness of the dataset, which may lead to overfitting. The model trained on the real-2 dataset achieved an *mAP* value of 95.66% on the validation set, surpassing the 94.80% and 95.39% *mAP* values obtained by models trained on the real-3 and real-4 datasets, respectively. This indicates strong performance on the validation set. However, on the test set, the *mAP* value dropped significantly to 65.15%, lower than the 66.00% and 67.44% *mAP* values achieved by models trained on the real-3 and real-4 datasets, respectively. The marked discrepancy between the validation and test set performance of the model trained on the real-2 dataset suggests overfitting, thereby substantiating this conclusion. On the other hand, when the data source of a real dataset is relatively single, models trained on this dataset exhibit poor generalization. Models trained on real datasets achieved lower *mAP* values on the validation set compared to those trained on mixed datasets, with a maximum difference of 4.05%. However, on the test set, the maximum difference increased to 10.7%. The larger discrepancy in *mAP* values on the more diverse test set demonstrates that models trained on small real datasets have poorer generalization capabilities.

(2) Feasibility of Synthetic Data as Training Datasets

The model trained on the synthetic dataset achieved an *mAP* value of 94.83% on the real-data validation set, which falls within the range of 93.32% to 95.66% for models trained on real datasets. This indicates that the model trained on synthetic data performs well, with a minimal gap compared to models trained on real data.

Moreover, the *mAP* value of the model trained on the synthetic dataset reached 71.84% on the test set, surpassing the highest *mAP* value of 67.44% achieved by models trained on real datasets. This demonstrates the feasibility and effectiveness of using synthetic datasets as training data for model development. However, it is important to note that training solely on synthetic data may lead the model to learn noise patterns that differ from those in real data, potentially degrading model performance.

(3) Advantages of Mixed Datasets

The models trained on the mixed dataset, which combines real and synthetic data, exhibit faster convergence, higher accuracy, and greater stability. Faster convergence: The training process illustrated in Figure 14 and Figure 16 demonstrates that models trained on the mixed dataset converge more rapidly compared to those trained solely on real or synthetic datasets. Higher accuracy: The models trained on the mixed dataset achieved *mAP* values ranging from 93.37% to 98.08% on the validation set and from 73.42% to 73.61% on the test set. These ranges are superior to the *mAP* values of models trained solely on real datasets, which ranged from 93.32% to 95.66% on the validation set and from 62.71% to 67.44% on the test set. Additionally, the mixed dataset models outperformed those trained solely on synthetic data, which achieved *mAP* values of 94.83% on the validation set and 71.84% on the test set. Greater stability: Figure 15 and Figure 17 show that as the proportion of real data in the mixed dataset increases, the *mAP* values of models trained on the mixed dataset improve more steadily and exhibit less fluctuation compared to models trained solely on real datasets. This highlights the enhanced stability of models trained on the mixed dataset.

Compared to both real and synthetic datasets, mixed datasets mitigated the shortcomings of limited real data and synthetic data noise, significantly enhancing model accuracy and generalization. However, the increase in the amount of real data in mixed datasets had a limited impact on model performance improvement.

## 5. Conclusions

### 5.1. Summary

In this study, we propose the VWP as an action detection data generator to overcome the challenges of insufficient data acquisition by visual sensors in real situations, acquisition difficulties, and single-source data in the field of action recognition in engineering production and operation, which are fields characterized by strong professionalism and high privacy. The VWP establishes a library of actions and behavior trees to control the actions and behaviors of virtual operators. It adds domain randomization to control various parameter changes and simulate multiple scenarios under different working conditions. Furthermore, it creates a data export pipeline to generate an action detection synthetic dataset in AVA format.

To verify the feasibility and effectiveness of the VWP-generated data, we introduced a spatiotemporal action detection method that uses YOLOv8 as the human detector, ViT-small as the backbone, and Slowfast as the action detection framework. Then, we collected datasets for the identification of safety risks in substations, comprising real-world data and synthetic data expanded through VWP domain randomization. Those datasets were categorized into three types: real datasets, synthetic datasets, and mixed datasets that combine both real and synthetic data. Furthermore, the real data were segmented into varying sizes to verify the impact of the real data volume on the model.

The synthetic datasets, real datasets, and mixed datasets were used to train the model, and real-world data were used as the test set to evaluate the model’s accuracy. The experimental results confirmed the feasibility, effectiveness, and robustness of using synthetic datasets to train models for spatiotemporal action detection in real-world scenarios. Furthermore, synthetic datasets can be augmented through domain randomization to include various perspectives, lighting conditions, and combinations of real and synthetic datasets, addressing the issue of single-source real data and effectively improving the *mAP* value of models of different sizes, with a maximum increase of 10.7%.

Subsequently, different datasets were used to train the models separately. To test the accuracy and generalization of models trained on different datasets, we used the model trained on a real dataset as the baseline and a mixed dataset of real data and AVA data as the test set. The training and testing results substantiated the following findings:

(1) When trained on smaller real datasets, the models’ performance was suboptimal, with a propensity for overfitting. The models derived from real datasets also underperformed in testing, indicating a lack of accuracy and generalizability.
(2) Synthetic datasets, expanded through domain randomization, encompass a diversity of data. The performance of models trained with synthetic datasets surpassed that of models trained on real datasets alone, affirming the feasibility and effectiveness of using synthetic data for model training.
(3) The mixed dataset, which was enriched with diverse synthetic data, mitigated the lack of diversity stemming from single-source real data, while the inclusion of real data alleviated noise issues associated with synthetic datasets. This combination effectively elevated the *mAP* values across different models, significantly enhancing their accuracy, generalizability, and stability.

In addition, VWP-generated synthetic datasets have other advantages compared to real datasets. In the VWP, action labels are annotated based on imported action animations with fixed start and end times, ensuring action label consistency and avoiding the issue of inconsistent time intervals when manually labeling actions. The VWP also has the advantages of easy operation, repeatability, parameterized modification control, and highly customizable editing. Of course, the VWP is only a virtual data synthesizer, and there are still differences between synthetic and real datasets, but the experimental results show that VWP’s synthetic datasets can partially replace and expand real datasets to a certain extent. Moreover, in this study, the VWP focuses on substation inspection operations as the research background; but in fact, the VWP can be extended to generate action recognition data for various fields, such as construction engineering, industrial production, and more.

### 5.2. Limitations

Although the VWP’s synthetic data can partially replace and expand real datasets, they still have some limitations. Firstly, virtual data cannot completely simulate the complex scenes and details of the real world. Secondly, the data generated by the VWP may be restricted by the specific model used. Finally, VWP-generated data may not cover all possible actions and scenarios, and therefore further development and improvement may be needed.

### 5.3. Future Work

To address the above limitations, future work can focus on the following tasks:

(1) Further improve the realism and diversity of VWP-generated data to better simulate complex scenes and details in the real world.
(2) Investigate how to apply VWP-generated data to a wider range of scenarios and tasks to expand its scope of application.
(3) Further expand the VWP’s action library and behavior trees to cover more actions and scenarios. Additionally, more data types, such as 2D and 3D data for key points of the human skeleton, could be added to the data export pipeline to support more types of action recognition algorithms.

## Figures and Tables

**Table 1:** Domain randomization parameters for datasets.

Category	Randomizer	Parameters	Distribution
Climate environment	Sun	Time	12, 17, 18, 19
	Weather	Sunny	/
		Rain and snow particle size	1–8
Shadows	Cloud	Shadow change rate	1–5
Animation	Animation playback	Playback speed	0.8–1.5
	Movement speed	Walk	1.2 m/s
		Run	1.7 m/s
Character position	Character	Movement area (walking or running)	4 × 10 m
		Character orientation angle	360° (15° rotate)
		Distance between character and camera	5 m, 6 m
Camera deployment	Camera	Camera angle (0° in front)	0°, 15°, 20°
		Camera height	1 m, 2 m, 3 m

**Table 2:** Computer configuration.

Computer	Category	Version
Hardware	GPU	NVIDIA GeForce RTX 2080 Ti 12 G
	Memory	62 G
Software	System	Ubuntu 18.04
	Programming language	Python 3.8.16
	Deep learning framework	Pytorch 1.11/torchvision10.2
	Compute Unified Device Architect	Cuda10.2/cudnn7.6

**Table 3:** Composition of datasets.

Dataset	Training Set	Validation Set	Test Set
Real-1	5 real videos (1 min)	5 real videos (1 min)	10 real videos (1 min) + AVA
Real-2	10 real videos (1 min)		
Real-3	15 videos (1 min)		
Real-4	20 videos (1 min)		
v	40 synthetic videos (5 min)	10 synthetic videos (5 min)	
Real-1 + v	5 real videos (1 min) + 40 synthetic videos (5 min)	5 real videos (1 min)	
Real-2 + v	10 real videos (1 min) + 40 synthetic videos (5 min)		
Real-3 + v	15 real videos (1 min) + 40 synthetic videos (5 min)		
Real-4 + v	20 real videos (1 min) + 40 synthetic videos (5 min)		

**Table 4:** Performance comparison of object detection models.

Model	*AP* (%)	Detection Time (ms/f)
RCNN	79.6	81.31
Faster R-CNN	89.2	131.4
RetinaNet	91.0	138.9
Yolov5	97.2	19
Yolov8	97.7	18.3

**Table 5:** Fine-tuning on real-4 and real-4 + v.

Optimizer	Real-4	Real-4 + v
Adam	0.86979	0.743856
Adamp	**0.94373** ^1^	**0.9730001**
Adamw	0.94204	0.972725
Nadam	0.83627	0.743663
Sgdp	0.50513	0.671545
Base learning rate	Real-4	Real-4 + v
1.00 × 10^−4^	0.87484	0.955812
2.50 × 10^−4^	0.92652	0.969106
5.00 × 10^−4^	0.94373	0.9730001
7.50 × 10^−4^	0.947231	0.977061
1.00 × 10^−3^	**0.949495**	**0.978911**
Layer-wise lr decay	Real-4	Real-4 + v
0.6	0.949495	0.978911
0.65	**0.953935**	**0.980849**
0.7	0.94499	0.978358
0.75	0.946856	0.976172
0.8	0.92348	0.978978

^1^ **Bold in gray** indicates the data that performs best under this hyperparameter compared to other hyperparameters.

**Table 6:** The *mAP* of the training datasets.

*mAP* (%)	Real	v
v	/	94.83
real-1	93.32	97.37
real-2	95.66	97.97
real-3	94.80	97.93
real-4	95.39	98.08

**Table 7:** The *mAP* of the test datasets.

*mAP* (%)	Real	v
v	/	71.84
real-1	62.71	73.41
real-2	65.15	73.55
real-3	66.00	73.40
real-4	67.44	73.61

## Data Availability

Data are contained within the article.

## References

[1] Amiri E., Sadeghi S.H.H., Moini R. (2012). A Probabilistic Approach for Human Safety Evaluation of Grounding Grids in the Transient Regime. IEEE Trans. Power Deliv..

[2] Shu Y., Tang Y. (2017). Analysis and Recommendations for the Adaptability of China’s Power System Security and Stability Relevant Standards. CSEE J. Power Energy Syst..

[3] Wei W., Tao Z. (2018). Occurrence and Countermeasures of Urban Power Grid Accident. IOP Conf. Ser. Earth Environ. Sci..

[4] Liu W., Wang X., Ye P., Jiang L., Feng R. (2023). Safety Accident Analysis of Power Transmission and Substation Projects Based on Association Rule Mining. Environ. Sci. Pollut. Res..

[5] Wang T., Wang K., Li J., Yu H., Shuai W., Bian J., Zhao X. (2017). Fast Recognition of Human Climbing Fences in Transformer Substations. Proceedings of the 2017 Ninth International Conference on Advanced Computational Intelligence (ICACI).

[6] Wang B., Ma F., Jia R., Luo P., Dong X. (2023). Skeleton-Based Violation Action Recognition Method for Safety Supervision in Operation Field of Distribution Network Based on Graph Convolutional Network. CSEE J. Power Energy Syst..

[7] Wang J., Zhou H., Sun H., Su Z., Li X. (2022). A Violation Behaviors Detection Method for Substation Operators Based on YOLOv5 and Pose Estimation. Proceedings of the 2022 IEEE 3rd China International Youth Conference on Electrical Engineering (CIYCEE).

[8] Kim H., Yi J.-S. (2024). Image Generation of Hazardous Situations in Construction Sites Using Text-to-Image Generative Model for Training Deep Neural Networks. Autom. Constr..

[9] Baik S., Kim E. (2025). Detection of Human Traffic Controllers Wearing Construction Workwear via Synthetic Data Generation. Sensors.

[10] Soomro K., Zamir A.R., Shah M. (2012). UCF101: A Dataset of 101 Human Actions Classes from Videos in The Wild. arXiv.

[11] Kay W., Carreira J., Simonyan K., Zhang B., Hillier C., Vijayanarasimhan S., Viola F., Green T., Back T., Natsev P. (2017). The Kinetics Human Action Video Dataset. arXiv.

[12] Kuehne H., Jhuang H., Garrote E., Poggio T., Serre T. HMDB: A Large Video Database for Human Motion Recognition. Proceedings of the 2011 International Conference on Computer Vision.

[13] Gu C., Sun C., Ross D.A., Vondrick C., Pantofaru C., Li Y., Vijayanarasimhan S., Toderici G., Ricco S., Sukthankar R. (2018). AVA: A Video Dataset of Spatio-Temporally Localized Atomic Visual Actions. Proceedings of the 2018 IEEE/CVF Conference ON Computer Vision and Pattern Recognition (CVPR).

[14] Goyal R., Kahou S.E., Michalski V., Materzynska J., Westphal S., Kim H., Haenel V., Fruend I., Yianilos P., Mueller-Freitag M. (2017). The “Something Something” Video Database for Learning and Evaluating Visual Common Sense. Proceedings of the 2017 IEEE International Conference on Computer Vision (ICCV).

[15] Dosovitskiy A., Ros G., Codevilla F., Lopez A., Koltun V. CARLA: An Open Urban Driving Simulator. Proceedings of the 1st Annual Conference on Robot Learning; PMLR.

[16] Shah S., Dey D., Lovett C., Kapoor A. (2018). AirSim: High-Fidelity Visual and Physical Simulation for Autonomous Vehicles. Field and Service Robotics.

[17] Faure G.J., Chen M.-H., Lai S.-H. (2023). Holistic Interaction Transformer Network for Action Detection. Proceedings of the 23rd IEEE/CVF Winter Conference on Applications of Computer Vision.

[18] Richter S.R., Hayder Z., Koltun V. (2017). Playing for Benchmarks. Proceedings of the 16th IEEE International Conference on Computer Vision, ICCV.

[19] Wrenninge M., Unger J. (2018). Synscapes: A Photorealistic Synthetic Dataset for Street Scene Parsing. arXiv.

[20] Tang Y., He H., Wang Y., Wu Y. (2024). Using a Diffusion Model for Pedestrian Trajectory Prediction in Semi-Open Autonomous Driving Environments. IEEE Sens. J..

[21] Tremblay J., To T., Birchfield S. (2018). Falling Things: A Synthetic Dataset for 3D Object Detection and Pose Estimation. Proceedings of the 31st Meeting of the IEEE/CVF Conference on Computer Vision and Pattern Recognition Workshops, CVPRW.

[22] Borkman S., Crespi A., Dhakad S., Ganguly S., Hogins J., Jhang Y.-C., Kamalzadeh M., Li B., Leal S., Parisi P. Unity Perception: Generate Synthetic Data for Computer Vision. https://arxiv.org/abs/2107.04259v2.

[23] Fabbri M., Lanzi F., Calderara S., Palazzi A., Vezzani R., Cucchiara R. (2018). Learning to Detect and Track Visible and Occluded Body Joints in a Virtual World. Proceedings of the 15th European Conference on Computer Vision, ECCV.

[24] Fabbri M., Braso G., Maugeri G., Cetintas O., Gasparini R., Osep A., Calderara S., Leal-Taixe L., Cucchiara R. (2021). MOTSynth: How Can Synthetic Data Help Pedestrian Detection and Tracking?. Proceedings of the 2021 IEEE/CVF International Conference on Computer Vision (ICCV 2021).

[25] Hu Y.-T.X., Chen H.-S., Hui K., Huang J.-B., Schwing A. (2019). SAIL-VOS: Semantic Amodal Instance Level Video Object Segmentation—A Synthetic Dataset and Baselines. Proceedings of the 2019 IEEE/CVF Conference on Computer Vision and Pattern Recognition (CVPR 2019).

[26] Hu Y.-T., Wang J., Yeh R.A., Schwing A.G. (2021). SAIL-VOS 3D: A Synthetic Dataset and Baselines for Object Detection and 3D Mesh Reconstruction from Video Data. Proceedings of the 2021 IEEE/CVF Conference on Computer Vision and Pattern Recognition, CVPR 2021.

[27] Ebadi S.E., Jhang Y.-C., Zook A., Dhakad S., Crespi A., Parisi P., Borkman S., Hogins J., Ganguly S. PeopleSansPeople: A Synthetic Data Generator for Human-Centric Computer Vision. https://arxiv.org/abs/2112.09290v2.

[28] Liu S., Ni H., Li C., Zou Y., Luo Y. (2024). DefectGAN: Synthetic Data Generation for EMU Defects Detection with Limited Data. IEEE Sens. J..

[29] Zhou G., Wang D., Yan Y., Chen H., Chen Q. (2022). Semi-Supervised 6D Object Pose Estimation without Using Real Annotations. IEEE Trans. Circuits Syst. Video Technol..

[30] Sharifi Renani M., Eustace A.M., Myers C.A., Clary C.W. (2021). The Use of Synthetic IMU Signals in the Training of Deep Learning Models Significantly Improves the Accuracy of Joint Kinematic Predictions. Sensors.

[31] Hou Y., Zhang S., Ma R., Jia H., Xie X. (2023). Frame-Recurrent Video Crowd Counting. IEEE Trans. Circuits Syst. Video Technol..

[32] Miller L., Navarro P.J., Rosique F. (2025). EdgeNet: An End-to-End Deep Neural Network Pretrained with Synthetic Data for a Real-World Autonomous Driving Application. Sensors.

[33] Krump M., Stütz P. (2023). Deep Learning Based Vehicle Detection on Real and Synthetic Aerial Images: Training Data Composition and Statistical Influence Analysis. Sensors.

[34] Sun Z., Ke Q., Rahmani H., Bennamoun M., Wang G., Liu J. (2023). Human Action Recognition from Various Data Modalities: A Review. IEEE Trans. Pattern Anal. Mach. Intell..

[35] Yeung S., Russakovsky O., Mori G., Li F.-F. (2016). End-to-End Learning of Action Detection from Frame Glimpses in Videos. Proceedings of the 2016 IEEE Conference on Computer Vision and Pattern Recognition (CVPR).

[36] Hou R., Chen C., Shah M. (2017). Tube Convolutional Neural Network (T-CNN) for Action Detection in Videos. Proceedings of the 2017 IEEE International Conference on Computer Vision (ICCV).

[37] Jain M., Van Gemert J., Jegou H., Bouthemy P., Snoek C.G.M. (2014). Action Localization with Tubelets from Motion. Proceedings of the 27th IEEE Conference on Computer Vision and Pattern Recognition.

[38] Oneata D., Revaud J., Verbeek J., Schmid C. (2014). Spatio-Temporal Object Detection Proposals. Proceedings of the Computer Vision—ECCV.

[39] Saha S., Singh G., Sapienza M., Torr P.H.S., Cuzzolin F. (2020). Spatio-Temporal Action Instance Segmentation and Localisation. Modelling Human Motion.

[40] Wang H., Schmid C. (2013). Action Recognition with Improved Trajectories. Proceedings of the 2013 14th IEEE International Conference on Computer Vision, ICCV 2013.

[41] Chen W., Corso J.J. (2015). Action Detection by Implicit Intentional Motion Clustering. Proceedings of the 2015 IEEE International Conference on Computer Vision (ICCV).

[42] Gemert J.C.V., Jain M., Gati E., Snoek C.G.M. (2015). APT: Action Localization Proposals from Dense Trajectories. Proceedings of the British Machine Vision Conference 2015.

[43] Gkioxari G., Malik J. (2015). Finding Action Tubes. Proceedings of the IEEE Conference on Computer Vision and Pattern Recognition, CVPR 2015.

[44] Saha S., Singh G., Sapienza M., Torr P.H.S., Cuzzolin F. (2016). Deep Learning for Detecting Multiple Space-Time Action Tubes in Videos. Proceedings of the 27th British Machine Vision Conference, BMVC 2016.

[45] Yang Z., Gao J., Nevatia R. (2017). Spatio-Temporal Action Detection with Cascade Proposal and Location Anticipation. Proceedings of the 28th British Machine Vision Conference.

[46] Ye Y., Yang X., Tian Y. (2019). Discovering Spatio-Temporal Action Tubes. J. Vis. Commun. Image Represent..

[47] Li Z., Gavrilyuk K., Gavves E., Jain M., Snoek C.G.M. (2018). VideoLSTM Convolves, Attends and Flows for Action Recognition. Comput. Vis. Image Underst..

[48] Vahdani E., Tian Y. (2022). Deep Learning-Based Action Detection in Untrimmed Videos: A Survey. IEEE Trans. Pattern Anal. Mach. Intell..

[49] Kalogeiton V., Weinzaepfel P., Ferrari V., Schmid C. (2017). Action Tubelet Detector for Spatio-Temporal Action Localization. Proceedings of the 2017 IEEE International Conference on Computer Vision (ICCV).

[50] Feichtenhofer C., Fan H., Malik J., He K. (2019). SlowFast Networks for Video Recognition. Proceedings of the 2019 IEEE/CVF International Conference on Computer Vision (ICCV 2019).

[51] Vaswani A., Shazeer N., Parmar N., Uszkoreit J., Jones L., Gomez A.N., Kaiser Ł., Polosukhin I. Attention Is All You Need. Proceedings of the Advances in Neural Information Processing Systems 30: Annual Conference on Neural Information Processing Systems 2017.

[52] Brown T., Mann B., Ryder N., Subbiah M., Kaplan J.D., Dhariwal P., Neelakantan A., Shyam P., Sastry G., Askell A. (2020). Language Models Are Few-Shot Learners. Adv. Neural Inf. Process. Syst..

[53] Han K., Wang Y., Chen H., Chen X., Guo J., Liu Z., Tang Y., Xiao A., Xu C., Xu Y. (2023). A Survey on Vision Transformer. IEEE Trans. Pattern Anal. Mach. Intell..

[54] Tong Z., Song Y., Wang J., Wang L., Koyejo S., Mohamed S., Agarwal A., Belgrave D., Cho K., Oh A. (2022). VideoMAE: Masked Autoencoders Are Data-Efficient Learners for Self-Supervised Video Pre-Training. Proceedings of the Advances in Neural Information Processing Systems 35 (NEURIPS 2022).

[55] Gonzalez-Franco M., Ofek E., Pan Y., Antley A., Steed A., Spanlang B., Maselli A., Banakou D., Pelechano N., Orts-Escolano S. (2020). The Rocketbox Library and the Utility of Freely Available Rigged Avatars. Front. Virtual Real..

[56] Carnegie Mellon University—CMU Graphics Lab—Motion Capture Library. http://mocap.cs.cmu.edu/.

[57] Tobin J., Fong R., Ray A., Schneider J., Zaremba W., Abbeel P. Domain Randomization for Transferring Deep Neural Networks from Simulation to the Real World. Proceedings of the 2017 IEEE/RSJ International Conference on Intelligent Robots and Systems (IROS).

[58] Ren S., He K., Girshick R., Sun J., Cortes C., Lawrence N.D., Lee D.D., Sugiyama M., Garnett R. (2015). Faster R-CNN: Towards Real-Time Object Detection with Region Proposal Networks. Proceedings of the Advances in Neural Information Processing Systems 28 (NIPS 2015).

[59] Lin T.-Y., Goyal P., Girshick R., He K., Dollár P. (2020). Focal Loss for Dense Object Detection. IEEE Trans. Pattern Anal. Mach. Intell..

[60] Jocher G. (2020). YOLO.

[61] Jocher G., Qiu J., Chaurasia A. (2023). YOLO.

[62] Terven J., Córdova-Esparza D.-M., Romero-González J.-A. (2023). A Comprehensive Review of YOLO Architectures in Computer Vision: From YOLOv1 to YOLOv8 and YOLO-NAS. Mach. Learn. Knowl. Extr..

[63] Sohan M., Sai Ram T., Rami Reddy C.V. (2024). A Review on YOLOv8 and Its Advancements. Proceedings of the Data Intelligence and Cognitive Informatics.

